# Experimental assessment of damage and microplastic release during cyclic loading of clear aligners

**DOI:** 10.1371/journal.pone.0318207

**Published:** 2025-02-05

**Authors:** Claudia Barile, Claudia Cianci, Vimalathithan Paramsamy Kannan, Giovanni Pappalettera, Carmine Pappalettere, Caterina Casavola, Michele Laurenziello, Domenico Ciavarella

**Affiliations:** 1 Dipartimento di Meccanica, Matematica e Management, Politecnico di Bari, Bari, Italy; 2 Dipartimento di Medicina Sperimentale e Clinica, Università di Foggia, Foggia, Italy; VIT University, INDIA

## Abstract

The widespread adoption of clear aligners in orthodontic treatments in recent years has necessitated a more precise examination of the mechanical properties of the devices currently available in orthodontics. Recent studies indicate that aligners, when exposed to the forces exerted during swallowing, undergo fatigue-like phenomena, leading to chip formation and cracks. The cumulative damage results in a compromised fit between the tooth and aligner, which is crucial for the effective execution of orthodontic treatment. Additionally, the formation of chips poses a potential risk to patients, as there is a possibility of inadvertently ingesting microplastics that become detached from the aligner over time. This study attempts to assess the release of microplastics from the aligners subjected to cyclic compressive loading. Three different aligners (Essix Ace, Ghost Aligner and Invisalign) are tested to simulate swallowing conditions over the aligner usage period. The mechanical performance is studied in terms of the energy absorbed by the aligner, which shows that the Essix Ace has a stable energy absorption behaviour, while the energy absorbed by the Invisalign is significantly higher than their counterparts. Ghost Aligner did not perform well in the cyclic compression tests. The microplastics (MPs) released by the aligners are examined under an optical microscope. A dimensional analysis based on k-means image segmentation and edge detection algorithm is developed to analyse the MPs. The dimensional analysis of the MPs revealed that the ingestion of the MPs released by all the three aligners does not pose a health risk.

## 1. Introduction

Over the last 25 years, Clear Aligner Treatment (CAT) has gained widespread popularity, replacing traditional fixed orthodontic appliances in many cases [1]. CAT involves the use of a series of aligners, custom-designed by orthodontists, to apply gradual movement forces on teeth to treat simple crowding or complex malocclusions [2–4].

The popularity of the CAT can be attributed to its aesthetic appeal among the adults, as the aligners used during treatment are virtually invisible in the oral cavity [2]. A survey made in the United States showed that 97% of the adult participants wanted to correct their malocclusion, but 62% of them were against using traditional treatments such as braces. Another similar survey in Sweden reported that 33% of the adults were against using traditional treatment [5]. Therefore, the CAT provides an aesthetic option for the adults undergoing treatment for the malocclusion. In addition, CAT is associated with improved periodontal health, a reduction in periodontopathic bacteria levels [6,7], a potential reduction in treatment time [8], and a reduction of soft tissue irritation.

While devices such as tooth positioners and spring aligners have been in widespread use since 1945, the clear aligners made their debut in 1998, introduced by Align Technology [2]. This development was increased by concurrent advancements in transparent thermoplastic materials and computer technology, notably Computer-Aided Design and Computer-Aided Manufacturing (CAD-CAM), together with sophisticated tooth-movement simulation software.

Since then, numerous companies have entered the clear aligner market, introducing innovative production techniques and materials to produce these devices. The manufacturing methods that have been studied extensively are thermoforming and additive manufacturing [9–11]. Researchers have studied the influence of thermoforming cycle and temperature on the dimensional stability of the pressure-formed and vacuum-formed aligners [9]. With respect to the additive manufacturing, the influence of printing parameters on the stability of the printed aligners was explored. Despite the constant advance s in this sector, published scientific investigations often struggle to keep pace, compelling orthodontists to rely on their clinical experience rather than on systematic studies [12]. The role of orthodontist, which is to elaborate the teeth movement planning without causing physiological damage becomes pivotal [13] as they navigate through various options in terms of materials, thickness, and production techniques, where each of these options could significantly impact the outcomes of the treatment.

The need to provide much information on the mechanical performance of different aligner configurations and to assist orthodontists in selecting the most suitable aligner for specific purposes remains a critical concern. This is particularly relevant given the aim to extend the use of clear aligner therapy to a broad spectrum of clinical cases, including malposition in children [14].

It is imperative to assess and demonstrate the aligner’s ability to maintain its dimensional and mechanical characteristics throughout its entire period of use. Recent studies have highlighted the tendency of clear aligners to undergo plastic deformation after two weeks of use, potentially compromising tooth-aligner fitting [10,11,15,16].

In addition, evidence of fatigue-like phenomena has been observed in aligners subjected to repetitive loads simulating swallowing activities. In these experiments, crack nucleation and chip formations in the aligners were observed [10]. These phenomena have two main adverse effects: the loss of the aligners’ mechanical characteristics and the potential for patients to swallow small plastic particles that may detach from the aligner. Currently, the latter effect is not adequately considered in clear aligner treatment planning [17].

A preliminary in-vitro study of orthodontic clear aligners from different manufacturers has highlighted the detachment of small plastic particles due to mechanical friction [18]. Quinzi et al. (2023) immersed the aligners in artificial saliva for seven days and stirred for five hours per day, simulating the physiological mechanical friction of teeth [18]. Although this research sheds light on this critical aspect of CAT, further investigations are required to comprehend the release of small plastic particles from aligners subjected to the effective stresses experienced during their use.

The term “microplastics” (MPs) was coined in 2004 to identify synthetic polymer particles or fibres with a diameter generally ranging from 1 to 5,000 μm [19–22]. The MPs are generally categorised into primary and secondary MPs. The primary MPs are the ones intentionally introduced into beauty and cosmetic products or added in the industrial additives. The secondary MPs are the ones detached from a material due to mechanical, physical, chemical or biological effects, A recent study by Tamargo et al. (2022) evaluated the risks associated with the presence of MPs in the digestive system [17]. They found that the ingestion of MPs alters the composition of the human microbial colonic community and theorized certain members of the colonic microbiota may adhere to the surface of MPs and promote biofilm formation.

In this study, the secondary MPs released during CAT were evaluated. (The secondary MPs will be referred simply to MPs from hereon.) An image segmentation algorithm based on k-means nearest neighbour algorithm is used to separate the MPs in the microscopic image for dimensional analysis. The novel method is developed based on the machine learning-based automatic and semi-automatic image segmentation algorithms used in medical and biomedical applications [23–27]. Further details of this method are provided in the subsequent sections. The experimental findings open the door to broader considerations regarding the duration of clear aligner treatment and the potential side effects associated with the ingestion of microplastics (MPs).

## 2. Materials and methods

Aligners were manufactured based on the original dental alignment of a subject with moderate crowding of upper and lower incisors, no prosthetic rehabilitation, fillings, or oral parafunctions. Digital dental alignments were acquired using an intraoral scanner (TRIOS-3Shape) at an acquisition rate of 1,875 images per second. The full arch scan took approximately 35 to 45 s. The images were acquired using the high-resolution acquisition mode, which is 31.5 microns. The dental alignment is processed using 3Shape OrthoAnalyzer® software to obtain a 3D reproduction of the dental arches.

Note that no human experiments were conducted on this study. The dental record of one of the authors, after consent, is used for manufacturing aligners. The ethics committee of the University of Foggia (42/CE/2019) approved the use of the dental record. This study is within the guidelines for observational studies of the Strengthening the Reporting of Observational Studies in Epidemiology (STROBE) [28]. All the procedures of this research protocol adhered to the Declaration of Helsinki of 1975, as revised in 2008.

All the aligners were programmed as passive aligners, precisely replicating the patient’s dental configuration and without inducing any tooth movement. The passive aligners are also called as post orthodontic retainer aligner (PORA), which is used at the end of the orthodontic treatment. Patients wear PORA for 22 hours to stabilize the malocclusion resolution and therefore, there is no teeth movement.

To this end, upper and lower casts in Daylight Hard Resin (Photocentric Ltd.) were fabricated from the 3D reproduction of the dental arches obtained by 3Shape OrthoAnalyzer®, using a Liquid Crystal HR2 (Photocentric Ltd.) 3D printer. The two casts were equipped with a central fin, allowing them to be gripped in the vices of the testing machine.

Subsequently, medical grade polyurethane discs (Ghost Aligner, Bart Medicals Ltd.) and PET-G discs (Essix ACE, Dentsply Sirona Inc.), each of thickness 0.75 mm, were thermoformed over the upper hard resin cast using the Erkodent® Erkoform 3D vacuum machine. The discs were heated by a medium wave infrared heater to 160 °C at a pressure of 0.8 bar. Then they were thermoformed by applying vacuum. The cooling time was 0.45 s.

The additively manufactured clear aligners of 0.5 mm thickness were supplied by Invisalign®, which internally produces all aligners necessary for treatment without providing information about materials and the production process. However, recent studies have indicated that these aligners are made of a blend of polyurethane [29].

To apply a mechanical load that simulates the occlusal force on the aligners, upper and lower hard resin casts were mounted on an INSTRON 3344 single-column universal testing machine, equipped with a 1 kN load cell.

During swallowing, an average human adult applies a 50 Nof load from the maxillary arch to the mandibular arch in an occlusal contact. In average, this action lasts for 1 second. Based on this, the cyclic test was designed [30–32]. Therefore, the 50 N load was selected to replicate the nominal occlusal force exerted by a human during the swallowing operation. For each test, a clear aligner sample was placed on the upper cast and the compressive load was applied. As the aligner was mounted on the dental casts, the occlusal contact between the aligner and the cast was achieved during the axial load. To achieve occlusal contact, the following procedure is followed. First, the lower cast was mounted on to the test machine with the pneumatic end grips and it was aligned horizontally using a spirit level. Then the clear aligner sample was attached to the maxillary arch of the upper cast. The upper cast was then rested on top of the lower cast to form an occlusal contact. Following this, the end grips were used to secure also the upper cast.

The aligners were placed in contact with artificial saliva during the cyclic compression tests to simulate the oral environment. Table 1 lists the chemical composition of the artificial saliva used in this study. The saliva used is a commercially available artificial saliva used for symptomatic treatment for patients with low salivary flow rate. The saliva is classified as a non-Newtonian fluid since it contains salivary glycoproteins. Animal mucin, polymeric thickening, moisturising agents like cellulose or water-soluble polymers are used as its replacement. The saliva used in this study contains 0.01% of Xanthan, which is an anionic, water-soluble biopolymer which exhibits low-shear viscosity and strong shear thinning properties similar to a non-Newtonian fluid. Xanthan is the key ingredient in artificial saliva that determines its viscoelastic behavior similar to human saliva. Xanthan has demonstrated high stability over a wide pH, temperature, and ionic strength range, as well as under shear. Its concentration is unlikely to change during the test, so the artificial saline maintained its physical properties even after the compression cycles [33–35]. The artificial saliva also contains both the organic and inorganic compounds, and the electrolytes present in the natural saliva. The main inorganic compounds are the chlorides of sodium, potassium and magnesium, which contributes to the ionic strength of the saliva. Phosphate (K_2_HPO_4_) and the organic compounds (sorbitol and E218) contribute to the buffering capacity of the saliva. In addition, the calcium chloride and the dipotassium phosphate also act as the remineralising compound in the saliva [36,37]. The pH level was kept neutral at less than 7.

**Table 1:** Chemical Composition of the Organic and Inorganic Components in the Artificial Saliva used to simulate Oral Environment.

Composition	Content (%)
Sorbitol*	4.3
KCl	0.12
NaCl	0.085
E218**	0.05
CaCl_2._ 2H_2_0	0.013
K_2_HPO_4_	0.013
MgCl_2_	0.005
Xanthan	0.01
Water	Balance

*Sugar Alcohol – C_6_H_14_O_6_; **Methylparaben – C_8_H_8_O_3_.

The procedure used in dentistry to simulate the oral conditions was followed in this study [37]. After mounting the aligner to the upper cast, a sponge impregnated in 15 ml of the artificial saliva was made in contact with the aligner. The sponge was cut so that it covered the aligner both internally and externally without affecting the dental occlusion. Then the upper and lower casts were securely held by pneumatically operated end-grips at a constant pressure of 10 MPa. To prevent the saliva from evaporating, the entire setup was closed by a cellulose hydrate film. In this way, the saliva was conserved during the entirety of the cycles.

The INSTRON Bluehill 2.5 software, used to control the testing machine, recorded displacement and force information at a sampling rate of 20 Hz. The tests were carried out at room temperature and under standard atmospheric conditions.

**Fig 1:**
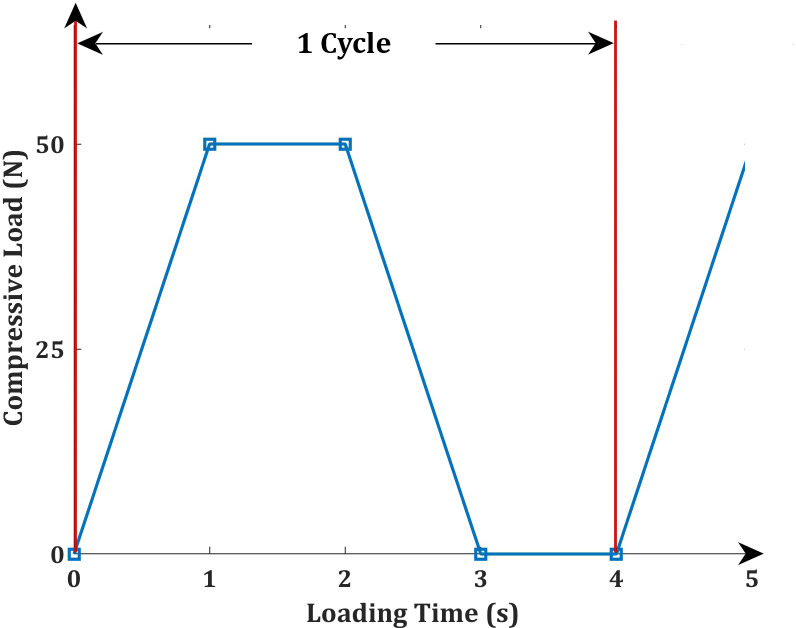
Four Step Compressive Loading Cycle used to Simulate the Occlusal Contact Force during Swallowing.

**Fig 2:**
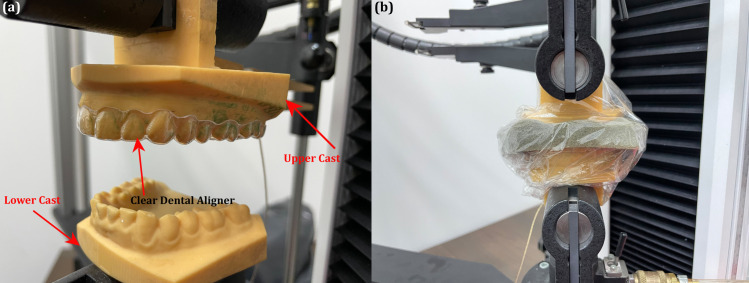
(a) Experimental setup of the upper and lower cast with the aligner and (b) the final test setup aligners in contact with the sponge soaked in artificial saliva and enveloped in cellulose hydrate filmimental setup of the upper and lower cast with the aligner and (b) the final test setup aligners in contact with the sponge soaked in artificial saliva and enveloped in cellulose hydrate film.

A loading cycle was performed 22,500 times, calculated based on the number of occlusal contacts during the swallowing act over the average aligner usage period. It has been reported that an average human swallows between 203 and 1,008 times per day [38,39]. Given this large variation in the number of swallows per day, 1,500 was considered as the nominal number of swallows per day and the cycles were repeated for the average aligner usage period of 15 days. Each load cycle had a duration of 4 seconds, distributed as follows (Fig 1): a compressive load ramped from 0 to 50 N in 1 second, followed by a dwell time of 1 second; then, a compressive load ramped down to 0 N in 1 second, with another dwell time of 1 second. The hold time during swallowing action, as reported in literature is 683 ± 249 ms. The maximum arbitrary hold time is 932 ms, which is rounded off to 1 second [40]. The hold time and the dwell time were also designed based on the occlusal contact during the swallowing [30]. The tests were performed at a constant cyclic load frequency of 0.25 cycles per second in a load-controlled mode (see Fig 2).

After each cyclic test, the cellulose hydrate film and the sponge were immediately removed, and any small drops of saliva present on the aligner were collected. These droplets were aspirated using an automatic pipette and transferred to a glass slide for subsequent microscopic observation. An inverted microscope, Axio Vert.A1 FL-LED (Carl Zeiss Microscopy, LLC), equipped with 20x and 40x magnification, was used to analyse the saliva slides. Images were captured using a CCD Camera mounted on the light microscopy system, utilizing Labscope v2.3 Software (Carl Zeiss Microscopy GmbH).

Furthermore, the inner surface of the clear aligners, which were properly cleaned with isopropanol (99% solution) to remove foreign particles, was examined using an optical microscope (NIKON SMZ800) both before and after the test. This analysis aimed to assess the effects of cyclic loading on each aligner.

## 3. Dimensional analysis of the microplastics

The microscopic images of the MPs in the glass slides are dispersed in the glass substrate together with the saliva. In order to separate the MPs for dimensional analysis, the images are segmented using an unsupervised image segmentation technique based on the k-means nearest neighbour algorithm [25]. In this segmentation process, the flattened RGB microscopic image are segmented into predefined number of clusters based on their features such as colour or texture attributes [24]. For an automated segmentation, it is essential to identify the optimal number of subjects, into which the image can be segmented. Cluster validity indices are commonly used to select the optimal number of clusters within an image. In this research wok, the Partition Entropy (PE) is used for autonomously select the optimal number of clusters. PE is one of the most efficient cluster validity indices used in k-means image segmentation. An optimal number of clusters should yield a high PE value.

### 3.1. k-means image segmentation

**Fig 3:**
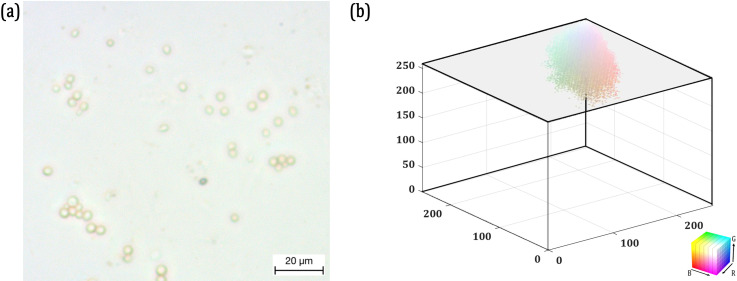
(a) A Sample Microscopic Image of the Artificial Saliva on the Glass Substrate; (b) The representation of the same Microscopic Image as RGB components in Feature Space.

For the k-means image segmentation, first the RGB image must be flattened into the feature space, as shown in Fig 3. Fig 3a shows a sample microscopic image taken for analysis and Fig 3b shows how the images is flattened into its RGB components and their distribution in the feature space. Now, the flattened image $X_{RGB}$ contains *n* datapoints $\left(X_{RGB}\;∋\;\left\{x_{1},\ldots ,x_{n}\right\}\right)$. Following this, the algorithm randomly assigns k number of centroids $C_{k}$, where k is the predefined number of clusters into which the image must be segmented. Then for each datapoint $x_{m}$, the Euclidean distance between the datapoint and the centroids $C_{k}$ are calculated. The datapoint $x_{m}$ is assigned to the closest cluster centroid. The objective of the algorithm is to assign the datapoints to the closest cluster centroid. Once all the datapoints are assigned to the clusters, then the cluster centroids $C_{k}$ are recomputed by taking the mean of the datapoints. Since the k-means image segmentation is highly dependent on the random assignment of initial centroids $C_{k}$, this process is repeated either until there is no change in $C_{k}$ between the successive iterations or a predefined number of iterations are reached. In this work, the process was repeated for 100 iterations. The $C_{k}$ was computed and monitored to ensure that there was no change in it between successive iterations at the end of the predefined 100 iterations [41].

### 3.2. Partition entropy

The optimal number of clusters *k*is selected using the Partition Entropy (PE). Entropy is the measure of the randomness or uncertainty of the data distributed in a dataset. In image processing, entropy quantifies the information content present of an image. In a clustered image, a higher entropy value returns the better quality in the segregation of pixel values sharing similarity [42–44]. The PE is more suitable for image segmentation with respect to other indices such as Silhouette or Davies-Bouldin, since it is suitable for images where the transition between the segments is smoother. The other indices are more suitable for images with hard boundaries and non-overlapping colour schemes.

The optimum cluster can be estimated using the PE, which is defined by Equation (1).


$$PE=-\frac{1}{n}\sum_{i=1}^{k}\sum_{j=1}^{n}\mu_{i,j}\;p\left(X_{i}\right)\ln\left[\mu_{i,j}\;p\left(X_{i}\right)\right]$$ (1)

$\mu_{i,j}$is the membership function of the datapoint *j* in the $i^{th}$ cluster. For the non-overlapping k-means clustering algorithm, the membership function $\mu_{i,j}$ is defined in Equation (2). $P(X_{i})$ is the probability of the occurrence of value *j* in the image *X*.


$$\mu_{i,j}=\left\{\begin{array}{c} 0,j\notin i\\ 1,j\in i \end{array}\right.$$ (2)

**Fig 4:**
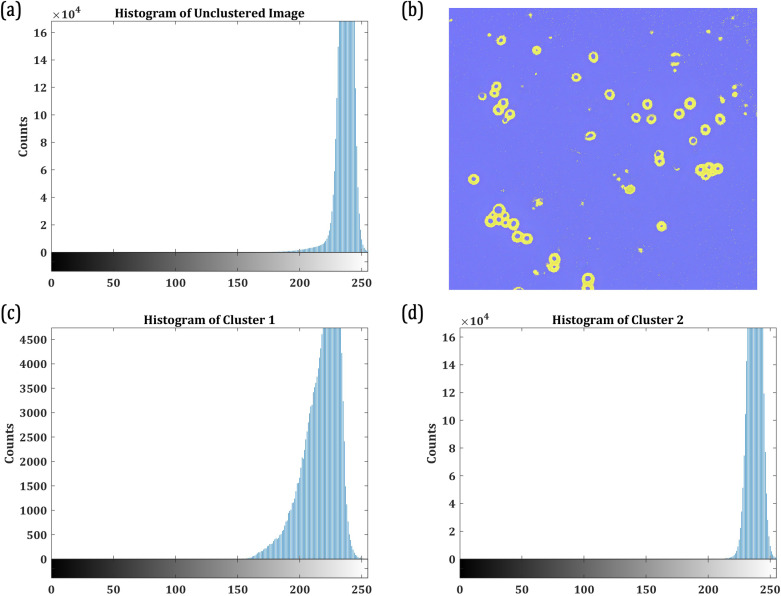
(a) Histogram of the Gray level Distribution in the Unclassified Microscopic Image (presented inFig 3a); (b) Clustered Image with k =  2; (c) Histogram of the RGB Distribution in Cluster 1; (d) Histogram of the RGB Distribution in Cluster 2.

Table 2 shows the PE for clusters k=2,3,…,6 for the sample microscopic image presented in Fig X1. PE returns the maximum for k =  2. Subsequently, the image is segmented into two clusters and is presented along with the partition histogram in Fig 4.

**Table 2:** Selection of Optimum Clusters for the Sample Microscopic Image using Partition Entropy.

Clusters	Partition Entropy
2	−0.6856
3	−0.8455
4	−0.7965
5	−0.9378
6	−0.9218

The histogram in Fig 4a represents the maximum occurrence of the pixels with colour intensities among the levels of neighbouring colour intensities. In principle, the entropies of the colour intensities in the clustered histograms (as in this case Fig 4c and 4d) quantify the uniformity in the distribution. The partition entropy returns the maximum value when the quality of information is rich in the classified clusters. In this example, as shown in Table 1, k =  2 is the optimum number of clusters into which Fig 4a must be segmented. Subsequently, the image is clustered and presented in Fig 4b.

Following this same procedure, all the microscopic images presented in this study are clustered based on their partition entropy and analysed further.

### 3.3. Edge segmentation and advanced image processing for dimensional analysis

**Fig 5:**
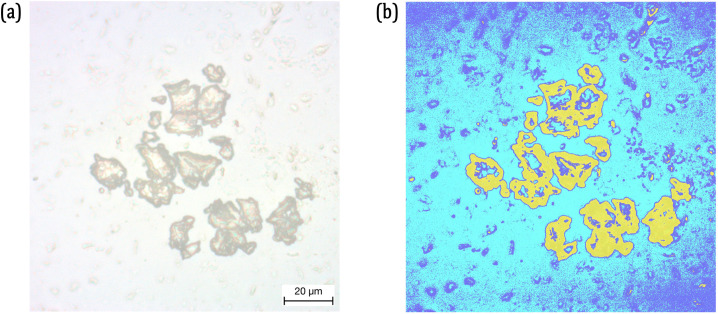
(a) Microscopic Image of a sample MP in the glass slide; (b) Image Segmented into 3 Clusters and the general shape of MP is highlighted in Yellow (Please refer to the Online Version of the article for the coloured image).

An image processing sequence is introduced in this research work to dimensionally analyse the objects in the microscopic image after segmentation. For this purpose, an example microscopic image containing MPs in the glass substrate with saliva is presented in Fig 5a. There are two reasons for not using the same example image as in Fig 3a for this section: 1) The microscopic image presented in Fig 3a is of the saliva without MPs and 2) To show the difference in image segmentation with and without MPs. Based on the PE, the microscopic image in Fig 5a is segmented into 3 clusters and the overlaid clusters are presented in Fig 5b.

After clustering, the general edge of the MP in Fig 5 can be visually identified. However, edge segmentation and region segmentation are necessary to extract the object of interest for analysis. The following steps are followed to extract the MP from the microscopic image.

Step 1:Cluster of interest is extracted from the clustered image.

Step 2:Separated Component is converted into grey scale by forming the weighted sum of the R, G and B Components.

Step 3:Grey scaled image is converted into black and white using a global threshold of 0.5.

Step 4:Sobel Edge detection operator is used to perform dimensional analysis on the object of interest in the image.

**Fig 6:**
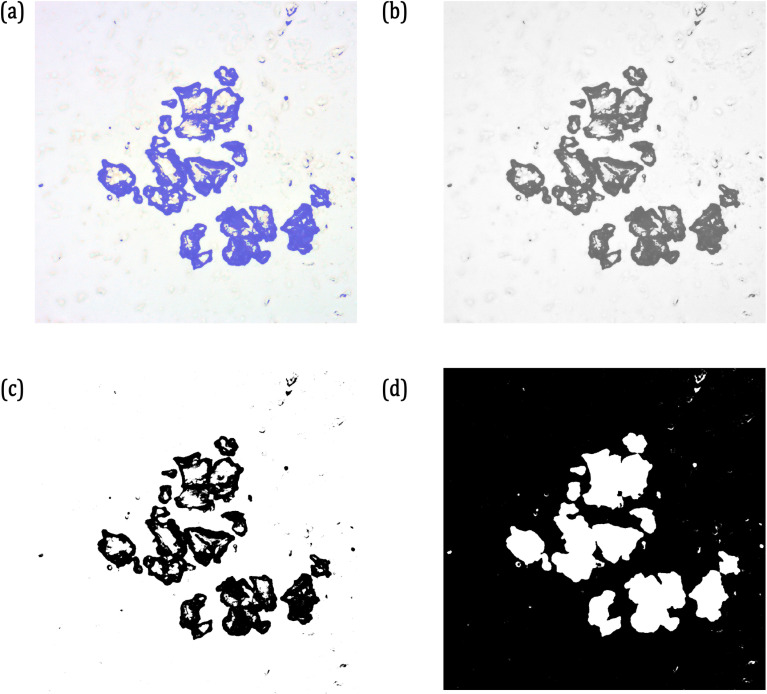
(a) Cluster of Interest Extracted from the Clustered Image; (b) Cluster of Interest converted into Grey Scale; (c) Grey Scale Image Converted into Black/White; (d) Object of Interest, which is the MPs separated using Edge Detection Operators.

This sequence is applied to the example image presented in Fig 5a and the results are presented in Fig 6. Two important factors need to be addressed following this image processing sequence. One is why the image cannot be directly converted into their grayscale instead of going through the clustering and two is why the image cannot be directly converted into black/white instead of following this sequence of operations. (For the interest of the readers, in S1 Appendix A, the images generated by directly converting the RGB image into black/white based on the threshold value and image clustered with a non-optimal cluster validity index is presented.)

Threshold-based segmentation is required when the target and the background have different levels of grayscale colour intensities. As can be seen from the feature maps and the histograms of the microscopic images presented respectively in Fig 4 and 5, the distribution of colour intensities and grayscale intensities are very close to each other. This requires the k-means image segmentation to extract the region of interest.

When converting an RGB image to black and white, the colour map is indexed to flatten the image to its grey scale before it is converted to black and white. Similar operations are performed sequentially in the proposed image processing sequence.

Following the image processing sequence, the dimensions of the particles are measured by Sobel edge detection method. It has been reported by several researchers that the Sobel edge detection operator is efficient in identifying the edges of both continuous and noncontinuous regions in biomedical images [23,25]. Multiple MPs present in the same microscopic images are dimensionally evaluated by 8-connected neighbourhood method. Both the Sobel-based edge detection and 8-pixel based connectivity are available in the Image Processing Toolbox of MATLAB^®^, which is implemented in this research work. The total number of pixels in the identified regions are MPs are used to calculate their total area, maximum dimension in the major and minor axes of the MPs.

To validate the accuracy of the dimensions calculated by the proposed method, for randomly selected microscopic images, the dimensions were also calculated manually. This was done in the Labscope v2.3 software, where the boundary nodes of the MPs are manually picked to create a contour and the area, major axis and minor axis of the MPs contour were calculated. The accuracy of this method depends on the precision of the node selection. However, there is a good correlation between the manually measured dimensions and the procedure used in this work. This procedure is cumbersome and redundant and therefore not included here.

The image processing routine introduced in this section is used to extract the MPs from the microscopic images and measure their area and lengths in the major and the minor axis. The results are presented and discussed in the subsequent sections.

## 4. Results

### 4.1. Mechanical test results

**Fig 7:**
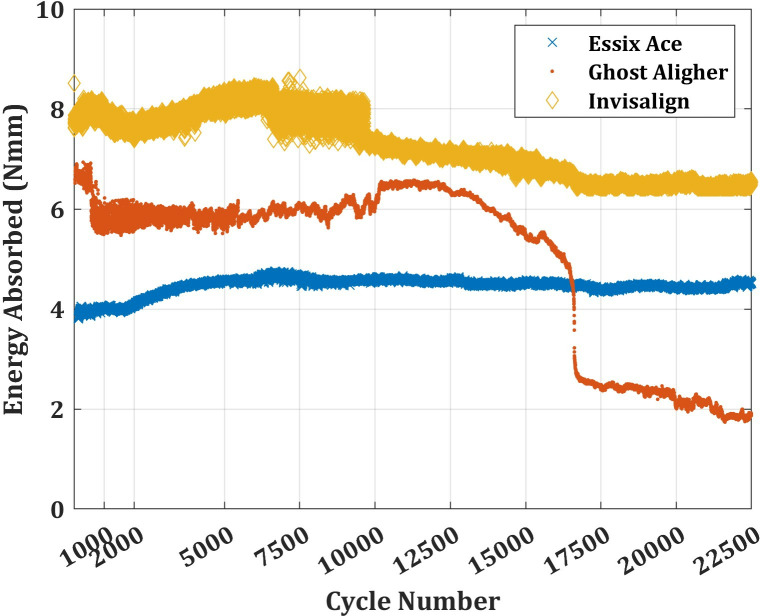
Energy Absorbed by the three different Clear Aligners under Cycle Compression Load up to 22,500 Cycles.

The mechanical test results are reported in terms of the energy absorbed by the aligners at different load cycles. The energy is calculated as the total area covered by the hysteresis curve obtained at each cycle different cycles. The results are presented in Fig 7 for the three aligners tested: Essix Ace, Ghost Aligner and Invisalign.

The average energy absorbed by Essix Ace is 4.45 ± 0.19 Nmm, which is significantly less than the Ghost Aligner (4.99 ± 1.67 Nmm) and Invisalign (7.26 ± 0.63 Nmm). However, the variance in the energy absorbed by Essix Ace over the 22,500 cycle is merely 0.0365, which is significant in this analysis. The Ghost Aligner absorbed an average energy of 6.03 Nmm with a variance of 0.11 up to cycle 12,000. Beyond this cycle, the amount of energy absorbed gradually decreases from 6.48 Nmm at cycle 12,001 to 6.62 Nmm at cycle 16,500, which is followed by a sudden drop to 2.68 Nmm at cycle 16,750. Beyond cycle 16,500, the amount of energy absorbed gradually decreases to an average of 2.29 Nmm with a large variance of 0.24 Nmm. Comparing this large variance with that of Essix Ace, one can see the stable energy absorption characteristics of the latter aligner.

In the case of Invisalign, there is a slight increase in the energy absorption between cycles 2,000 and 5,500, where it increases from 7.44 Nmm to 8.11 Nmm with a variance of 0.49. From cycle 5,500 to cycle 10,000, it fluctuates considerably with a variance of 0.14 Nmm, while the average energy absorbed remains at 7.90 Nmm. The energy absorption then decreases quite gradually from 7.90 Nmm to 7.18 Nmm at cycle 17,000 with a variance of 0.42. It then remains stable until the end of the test with a very small variance of 0.0015 Nmm.

Each aligner shows different energy absorption characteristics since they differ either in their base material or in their manufacturing process. Essix Ace shows stable but low energy absorption compared to the other two aligners which are made from PU. It is well-known that PU is characterised by large strain deformations compared to the more rigid PET-G [45], which is the base material for Essix Ace. Consequently, during deformations, PU aligners tend to absorb more energy compared to PET-G. This is evident in Fig 3, where the PU-based aligners Ghost Aligner and Invisalign absorbed more energy than PET-G. The sudden drop in energy in the Ghost Aligner can be explained by the sudden release of the absorbed energy as a consequence of damage evolution. Although Invisalign did not exhibit a significant drop in the absorbed energy, the drop after cycle 10,000 is noteworthy. To observe this damage evolution, two modes of microscopic analysis is carried out in this study.

### 4.2. Dimensional analysis of the microplastics

Firstly, the residual saliva is collected from the tested aligners using an automatic pipette and placed between two glass slides and observed under the microscope. Secondly, the tested aligners are inspected under an optical microscope (NIKON SMZ800) before being cleaned with isopropanol.

**Fig 8:**
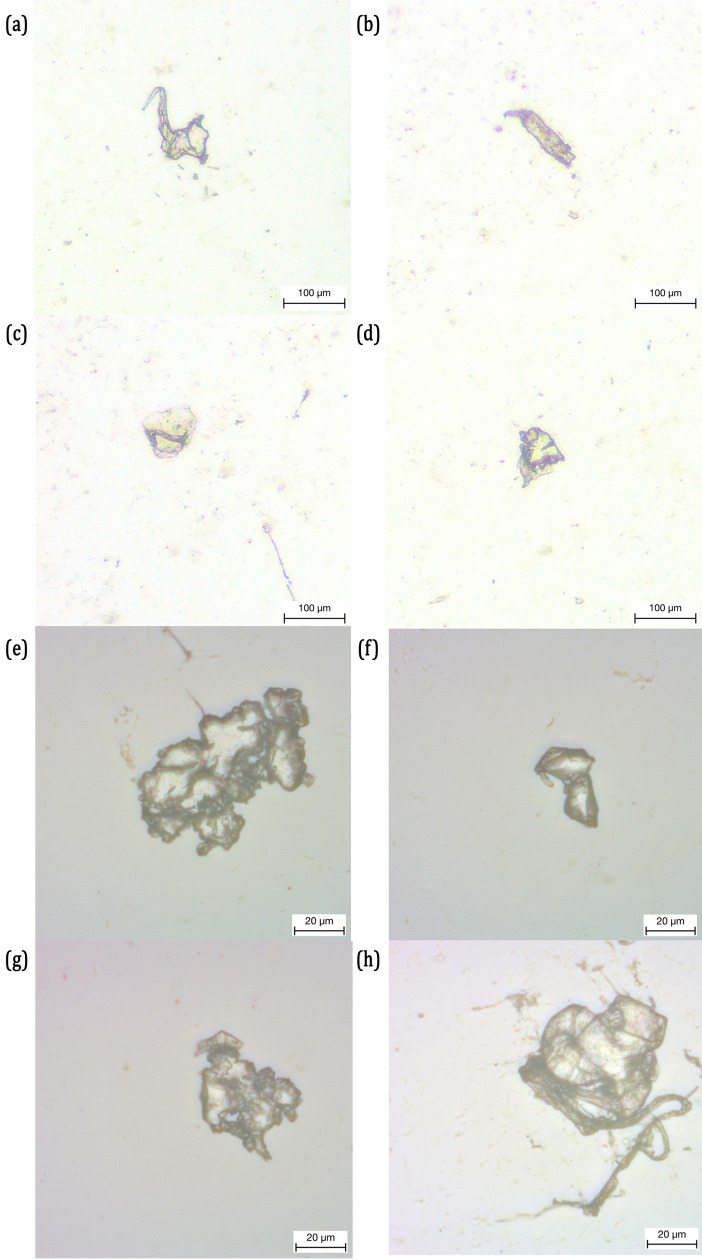
Microscopic Images of the Microplastic Particles in the glass slides, released from Essix Ace and acquired from the Residual Saliva.

Fig 8 presents the microscopy images, captured at 10x and 40x magnification, of the saliva collected immediately after the test on the Essix Ace aligner. Using the image processing routine proposed in Section 3 is used to calculate the area, maximum major axis length and minor axis length of the MPs presented in Fig 8. The results are presented in Table 3.

**Table 3:** Dimensions of Microplastic Particles released from Essix Ace during the Cyclic Compression Test.

Figure Number	Area	Major Axis Length	Minor Axis Length
	$\mu m^{2}$	μm	μm
8a	1,225.76	62.34	35.49
8b	684.62	56.34	14.47
8c	591.34	33.39	23.75
8d	700.15	34.70	26.91
8e	5,097.38	111.82	61.56
8f	864.91	51.22	25.70
8g	2,116.66	65.14	46.28
8h	3,055.08	67.22	63.06

Despite the energy absorption characteristics of Essix Ace remain stable, several MPs can be observed from the collected residual saliva. All the MPs in Fig 8 detached from Essix Ace appear to be irregular fragments with different shapes and sizes. Their dimensions do not seem to be regular or uniform; the MP with largest area is 5,097 $\mu m^{2}$ in Fig 8e while the one with the smallest area is 685 $\mu m^{2}$ in Fig 8c. The same irregularity is observed also in their dimensions where the major and minor axis lengths have a wider distribution. The major axis length of the MPs ranges from 30 μm to 65 μm in general, while the MP in Fig 8e has major axis length greater than 111 μm. The minor axis length ranges between 14 μm and 64 μm.

**Fig 9:**
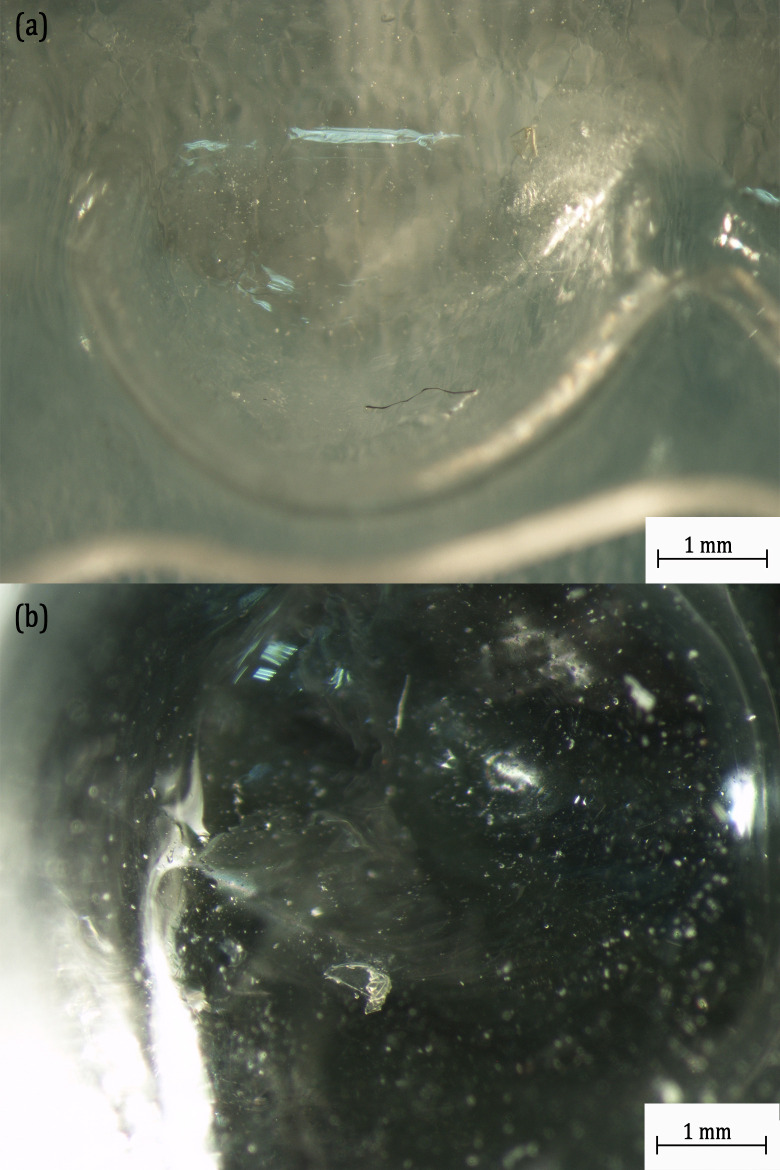
Optical Microscopic Images of Essix Ace Aligner at different Tooth Positions: (a) First Premolar on the Upper Right showing Crack Formation; (b) Second Molar on the Upper Left showing Chip Removal.

In Fig 9, the optical microscopic analysis on the tested Essix Ace aligner revealed microcracks in the first premolar on the upper right dental position and a small chip of material removed from the second molar on the upper left. (For the tooth denomination, please refer to S2 Appendix B). These microcracks and chip removal are some of the possible sources for the detachment of MPs from the Essix Ace aligner.

**Fig 10:**
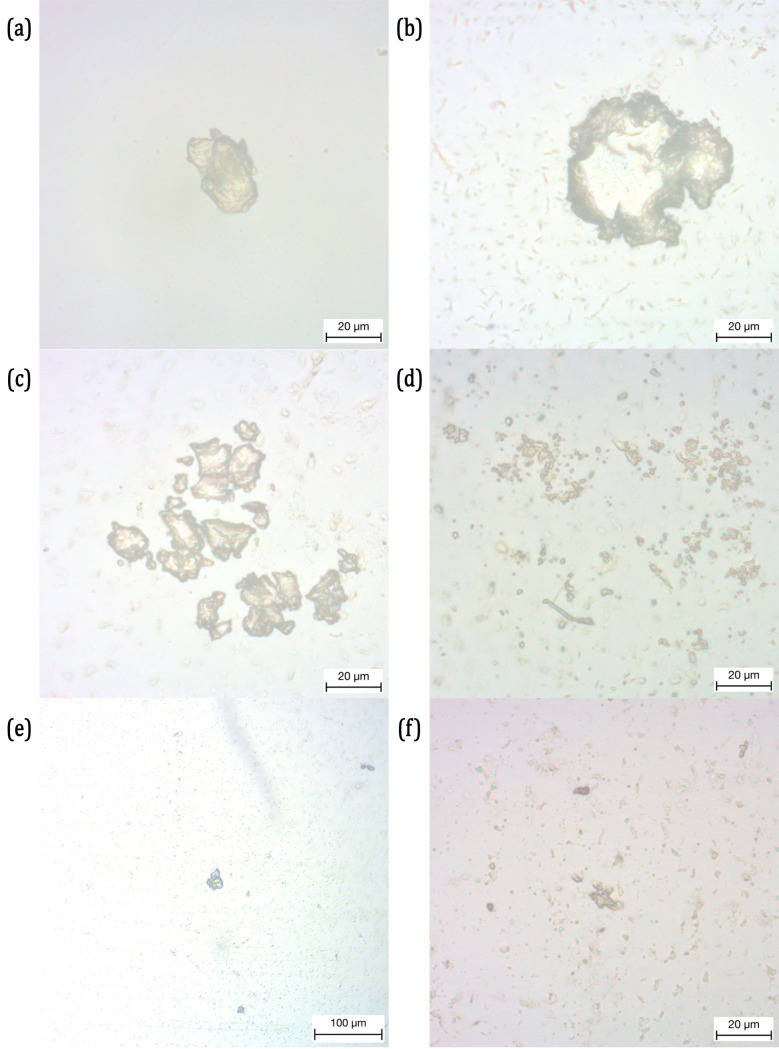
Microscopic Images of the Microplastic Particles in the glass slides, released from Ghost Aligner and acquired from the Residual Saliva.

Similarly, the MPs obtained from the Ghost Aligner are presented in Fig 10 and their dimensions are calculated and presented in Table 4. The area of the MPs detached from the Ghost Aligner ranges roughly from 180 $\mu m^{2}$ to 4,400 $\mu m^{2}$. The MP in Fig 10b has the largest area while the smallest is in Fig 10f. In Fig 10c, there are several fragments of the MP present in the micrograph. Therefore, the average dimensions are provided in Table 4. The total area of the particles present in the micrograph is 3,931 $\mu m^{2}$. For Fig 10d, the dimensions of the particles are indistinguishable, therefore, their total area in the micrograph is presented. Similar to the case of Essix Ace aligner, the MPs detached from Ghost Aligners are mostly of irregular shape and nonuniform dimensions. However, the MPs in Fig 10a and 10b have a regular shape. The MP is more or less elliptical in shape in Fig 10a with a major axis length of 45 μm and a minor axis length of 29 μm, while circular with a diameter roughly ranging between 81 μm and 72 μm in Fig 10b. The dimensions of the MPs in Fig 10c, although is indistinguishable using the procedure introduced in Section 3, the appear to have dimensions in at least one of their principal axes less than 1 μm. Although very few MPs are recovered from the tested Ghost Aligner, their dimensions in major axis and minor axis are slightly less than those recovered from Essix Ace aligner.

**Table 4:** Dimensions of Microplastic Particles released from Ghost Aligner during the Cyclic Compression Test.

Figure Number	Area	Major Axis Length	Minor Axis Length
	$\mu m^{2}$	μm	μm
10a	1,011.41	44.67	28.78
10b	4,383.42	80.72	71.51
10c^*^	652.08	34 ± 9.35	24 ± 9.03
10d	2,255.40	–	–
10e	1,256.21	39.81	7.49
10f	179.65	17.29	10.07

* Corresponds to the average dimensions.

**Fig 11:**
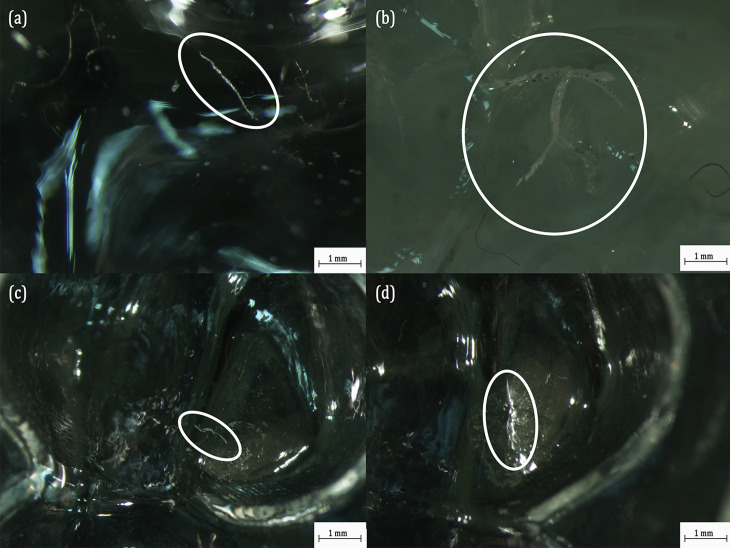
Optical Microscopic Images of Ghost Aligner at different Tooth Positions: (a) First Molar on the Upper Right showing Crack Formation; (b) Second Molar on the Upper Right showing Wider Cracks; (c) Second Premolar on the Upper Right Showing Strain Hardened Regions and Cracks; (d) Second Premolar on the Upper Left Showing Strain Hardened Regions.

Similar to the previous case, the optical microscopic analysis is carried out on the Ghost Aligner post testing and the results are presented in Fig 11. Unlike in the case of Essix Ace aligner, several microcracks from different tooth positions are observed in Ghost Aligner. Several microcracks can be observed in the first molar on the left (Fig 11a), second premolar on the right (Fig 11c and second premolar on the left (Fig 11d). Most of these cracks have lengths around 1 mm to 1.5 mm. However, on the second molar on the left in Fig 11b, several branches of crack originating from the apex crown position of the second molar on the right side are observed. These cracks have lengths exceeding 3.5 mm in length. These are some of the potential sites from which the MPs are detached during the compression test. It must be noted that all the MPs detached from the aligners are recovered for the dimensional analysis. The MPs recovered from the residual saliva collected after the compression test are the ones analysed. The presence of several cracks and severity in the damage is consistent with the results of compression test, where the energy absorbed by the Ghost Aligner reduced significantly beyond 16,750 cycles. The energy absorbed by the Ghost Aligner is in fact released during the crack growth. This may also have led to the detachment of several MPs from this aligner.

**Fig 12:**
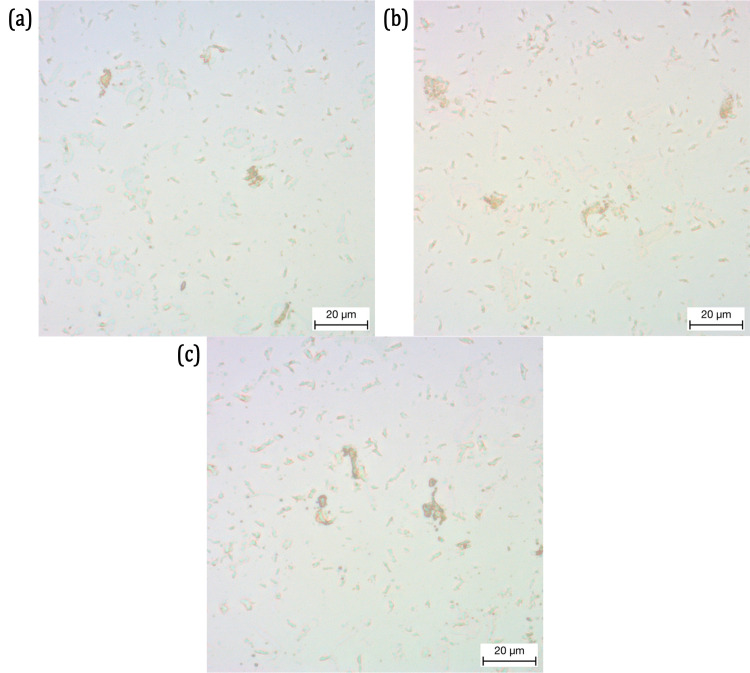
Microscopic Images of the Microplastic Particles in glass slides, released from Invisalign and acquired from the Residual Saliva.

The microscopic images of MPs obtained from testing Invisalign are presented in Fig 12. The dimensions of those MPs are presented in Table 5. Both the areas and the major and minor axis lengths corresponds to the average dimensions of the MPs present in Fig 12. Very few detached MPs are recovered from Invisalign after the compression test. The average area of the MPs in Fig 12a and 12c are roughly around 150 $\mu m^{2},$ while the MPs in Fig 12b is 440 $\mu m^{2}.$ Similar to both the previous aligners, the MPs are irregularly shaped with nonuniform dimensions. Their dimensions, however, are the smallest compared to the other two aligners. The major axis dimensions are roughly between 12 μm and 23 μm, while their minor axis dimensions are between 6 μm and 10 μm.

**Table 5:** Dimensions of Microplastic Particles released from Invisalign during the Cyclic Compression Test.

Figure Number	Area	Major Axis Length	Minor Axis Length
	$\mu m^{2}$	μm	μm
7a	153.32	12.02 ± 2.87	6.33 ± 1.40
7b	441.05	20.89 ± 5.22	9.15 ± 1.47
7c	144.29	16.05 ± 4.98	7.20 ± 3.16

**Fig 13:**
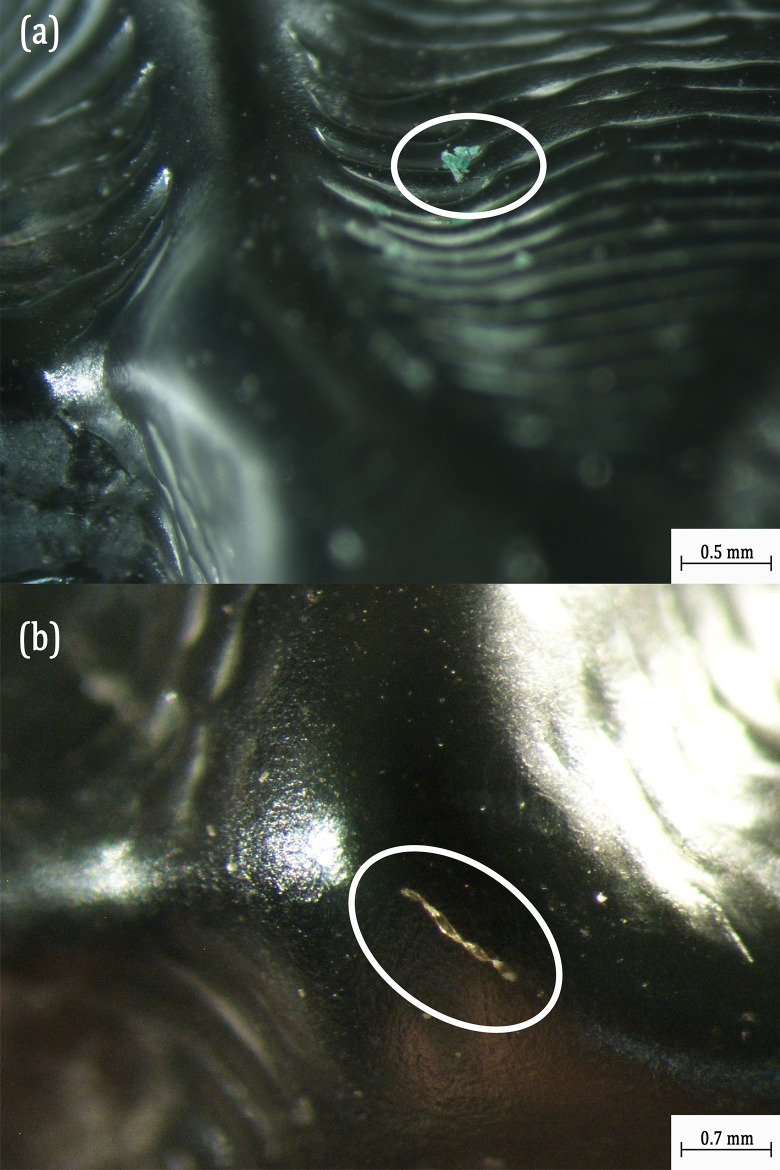
Optical Microscopic Images of Invisalign at different Tooth Positions: (a) Canine on the Upper Left showing Chip Removal; (b) Second Molar on the Upper Left Showing Crack Formed.

The optical microscopic images of Invisalign are presented in Fig 13. Very few damaged regions are observed in this aligner. In particular, a chip is removed in the upper left canine (Fig 13a) and a crack of length approximately 1 mm is found in the second molar on the upper left (Fig 13b).

### 4.3. ANOVA analysis

One-way ANOVA test is carried out to determine whether the dimensions of the MPs released by the clear aligners differ significantly. The results are summarised in Table 6. The area, major axis and minor axis lengths of the MPs released by Essix Ace and Ghost Aligner were tested. When tested at a 5% significance level, the one-way ANOVA test yielded p =  0.84, 0.24, and 0.48 for the area, major axis length, and minor axis length, respectively. The p-values are much larger than the 5% significance level (α=0.05) for all three dimensions and thus, fail to reject the null hypothesis. This indicates that there is no significant difference between the dimensions of the MPs released by Essix Ace and Ghost Aligner.

**Table 6:** One-way ANOVA Test Results to compare the Dimensions of the MPs.

Comparison	p-value		
	*Area*	*Major Axis Length*	*Minor Axis Length*
Essix Ace/Ghost Aligner	0.84	0.24	0.48
Invisalign/Essix Ace & Ghost Aligner	0.12	0.04*	0.02*

*p <  0.05 is considered statistically significant.

The grouped dimensions of the Essix Ace and Ghost Aligner are compared with that of the Invisalign. The ANOVA test yielded a p-value of 0.12 for the area of the MPs released, which indicates that there is no significance difference between these aligners. However, the p-values for major axis and minor axis lengths are 0.04 and 0.02, respectively, which rejects the null hypothesis. This indicate that there is a significant difference between the major and minor axis lengths of the MPs released from the Invisalign and the Essix Ace and Ghost Aligner.

The apparent differences and similarities of the dimensions of the MPs released by the three tested clear aligners and their importance are discussed in the next section.

## 5. Discussion

The detachment of MPs is apparent from all three aligners tested in this study. In the literature, the detachment of MPs from the aligners during the CAT is expected to be a result of the mechanical friction. However, this study reveals that the MPs are detached as a result of different damage progressions such as crack growth and chip formation from the aligners during the compressive cyclic loading. It is worth mentioning that the cyclic compression test was designed to simulate load effects of swallowing events during the usage of the aligners over 15 days of CAT. As each of these aligners undergo different deformations and damage progressions over the 22,500 simulated swallowing cycles, the possibility of detachment of MPs and their ingestion is evidently inevitable. Evidently, the Ghost Aligners performs poorly under the compressive cyclic test, where large number of cracks are observed at different dental positions. In a recent study by Lümkemann et al. (2023) [9], it was found out that the dimensional stability and reproducibility of the thermoformed aligners are poor compared to the additively manufactured ones. Irrespective of the thermoforming cycle, the dimensional reproducibility always remains poor for the thermoformed aligners. In addition, thermal stress is induced on these aligners during cooling. A not perfect dimensional correspondence in Ghost Aligner might have caused mechanical friction with the die cast and consequently the MPs are detached. The thermal stress must have caused localized stress zones, which resulted in the propagation of microcracks and ultimately the failure of the aligner. This is evident from the energy absorption curve, where the amount of energy absorbed dropped from 4.90 Nmm to 2.68 Nmm at cycle 16,750. The severity of the damage in the Ghost Aligner and different damage zones are potential detachment sites for the MPs. The residual MPs collected from the Ghost Aligner have wider size and area distribution.

Although Essix Ace is a thermoformed aligner, it does not exhibit the same damage characteristics as the Ghost Aligner. As already mentioned in the Section 4.1., these aligners differ in their base material. PET-G, the base material for Essix Ace is more rigid under compression compared to PU-based Ghost Aligner. Nonetheless, the detachment of MPs is also observed in Essix Ace. In fact, a large number of residual MPs are collected from this aligner. Interestingly, most of the MPs have major axis length greater than 30 μm, while in Ghost Aligner MPs with size less than 30 μm are frequently observed. In fact, the ANOVA test results show that there is no significant difference between the area, major axis length, and minor axis length of the MPs released from these aligners. This raises a question whether the size of the MPs is material dependent.

Looking at the Invisalign, the detached MPs have major axis and minor axis length generally less than 30 μm. More interestingly, a very few residual MPs are collected from the Invisalign. It was already mentioned that not all the detached MPs are collected from the saliva after the test. Nonetheless, the size distribution of the MPs and the number of MPs collected from Invisalign is significantly smaller than that of the other two aligners. This is also validated by the ANOVA test. While comparing the Invisalign with the Essix Ace and Ghost Aligner, the p-values of the major axis and minor axis lengths are less than 0.05 indicating a significant difference between them. In the study by Lümkemann et al. (2023) [9], it was explained that the additively manufactured aligners have more dimensional reproducibility compared to the thermoformed ones. Therefore, the Invisalign possibly underwent less mechanical friction compared to the others. The microscopic results showing very few damage zones and reasonable stability in the energy absorption characteristics concur this observation. Since the dimensions of the MPs detached from the PU-based Invisalign is less than 30 μm, and the p-values of 0.04 and 0.02 for the major and minor axis lengths, the size distribution of the MPs can be deemed material dependent.

Why is it important to discuss about the size distribution of MPs and why is it necessary to develop a dimensional analysis procedure to accurately estimate the dimensions of the MPs? In recent years, the ingested MPs have become a novel pollutant due to their ubiquitous distribution and toxicity [18]. Depending on their size, MPs can cross cell membranes. Particularly, the MPs of dimensions less than 1 μm can passively pass through cell membranes [46]. In addition, if the MPs are of thin-fibre shaped causes cell shape perturbations, whereas the interaction is less severe if they are ingested as fragments or microspheres [47].

Recent research works have reported on the primary health effects of ingested MPs. Digestive irritation or intestinal dysbiosis are reported to be caused by the ingested MPs [17]. Depending on the shape and size of the MPs they may pass through the digestive tract and cause serious health problems. At gastrointestinal level, while smaller MPs can pass through digestive tracts passively, most of the MPs between sizes 5 μm and 20 μm may not pass through epithelium passively. However, they may pass through either by endocytosis mechanisms or by paracellular diffusion [48,49]. It was reported that the MPs pass through the endocytosis may be reach the circulatory system through the translocation by dendritic cells through lymphatic circulation. On the other hand, the ingested MPs of size greater than 20 μm are likely to be excreted from the gastrointestinal tract.

The ingested MPs in the circulatory system may exhibit cytotoxicity characteristics. This is commonly associated with the monomer leach from the MPs. The monomer leach of the MPs is strongly associated with the manufacturing method of the aligner. It has been reported by several researchers that the increase in temperature during the thermoforming process is one of the primary reasons for the monomer leach [50,51]. This, however, is absent in additively manufactured aligners when they are fabricated through stereolithography [52–56]. Some researchers have done accelerated in-vitro aging of additively manufactured aligners and quantitatively characterised the leached substances through gas chromatography mass spectroscopy (GS-MS). The results show that no traceable monomers are found, and the risk of cytotoxicity is less in the usage of these aligners [57,58]. While testing at varying time intervals, the additively manufactured aligners show traces of cytotoxicity during the initial days use, and it decreases as the time progresses. Nevertheless, the level of cytotoxicity is still very low [29,53,59].

In this study, most of the MPs collected from Essix Ace and Ghost Aligner have dimensions greater than 20 μm. Therefore, it is safe to assume that most of them will be excreted from the gastrointestinal tract. Therefore, the risk of cytotoxicity is avoided while ingesting the MPs from these two aligners. The MPs collected from Invisalign are less than 20 μm in size, which means that any potential health hazards due to their ingestion require further investigation. Invisalign aligners are manufactured through stereolithography, which means that the monomer leach from the ingested MPs of this aligner is less likely [55,56].

All the three aligners tested are potentially safe to use although there is a strong possibility that the detached MPs are ingested during the CAT. Since most of the collect MPs from Essix Ace and Ghost Aligner have sizes greater than 20 μm, they are possibly excreted from the gastrointestinal tracts. Despite the size of MPs from Invisalign is less than 20 μm, the risk cytotoxicity from the ingested MPs is very low and the aligner can be safely used.

However, the growing scientific evidence shows that human intakes 39,000 to 52,000 MPs through ingestion every year [60]. Considering this, the ingestion of MPs due to the CAT cannot be ignored. Although there is no evidence on widespread human health risk due to the ingestion, the scientific community has raised an alarm to understand the human exposure to MPs [49,61–63].

The present study reveals that despite the detachment of MPs as fragments from the clear aligners, they are potentially safe for CAT. It must be noted that this study did not account for the effects of body temperature, oral humidity and other salivary enzymes on the CAT.

### 5.1 Limitations and future studies

The present study did not consider the effects of body temperature, oral humidity or the other salivary enzymes on the CAT. Very few research works are available in the literature which discusses the adverse effects of the salivary enzymes to the clear aligners. The future remains open for expanding this study to understand the effects of the salivary enzymes.

Although the cytotoxicity of the released MPs is discussed in the previous section, their biological properties remain unknown. In most literature, in-vitro experiments are carried out to analyse the cytotoxicity of the leached MPs from the aligners, but it does not take into account the fact that the MPs have high surface-to-volume ratio, which may increase their biological activity with the human salivary enzymes or gastrointestinal fluids. More quantitative analysis of the cytotoxicity of the MPs must be addressed in the future.

Furthermore, in this study the MPs are collected at the end of the cyclic tests and analysed. This study is proposed to be extended to collecting all the MPs detached from the CAT, in order to present a comprehensive analysis. Future developments include carrying out analysis on the detached MPs at constant time intervals, to evaluate the dimensional trend of the MPs as a function of time and to evaluate the total number of particles. In addition, the mechanical performance of the biocompatible aligners needs to be evaluated to overcome the problems involving the ingestion of MPs during the CAT.

## 6. Conclusions

In this study, the mechanical behaviour of three clear aligners, Essix Ace, Ghost Aligner and Invisalign, under simulated swallowing conditions was studied for 22,500 cycles. The mechanical performance showed that Essix Ace exhibited stable energy absorption characteristic compared to their counterparts. Ghost Aligner performed poorly as the amount of energy absorbed dropped after 16,750 cycles. Invisalign, although absorbing higher energy than Essix Ace, still had large variance in its behaviour between 10,000 and 17,000 cycles. The mechanical stability and application of Invisalign is extensively investigated in literature. Although most of these results are in-vitro studies, the advantages of Invisalign over conventional orthodontic treatments are well-known. The results from the present study suggest that Essix Ace, from a mechanical perspective, could outperform Invisalign due to its stable energy absorption characteristics over the 22,500 cycles. Nevertheless, in future works, several test samples must be analysed to present statistically significant findings.

In addition to the mechanical behaviour, the detachment of MPs from the aligners under these cyclic loading conditions and the health risks associated with their ingestion were investigated. The results showed that a large number of MPs are detached from all the three aligners. In general sense, the average size of the MPs released from Essix Ace and Ghost Aligners possibly are sufficiently large enough to be excreted through the gastrointestinal tract. Nevertheless, the shape effects, the biological reaction due to the high surface-to-volume ratio of these MPs and the effects of their extensive usage period needs to be investigated. On the other hand, the size of the MPs detached from the Invisalign poses risk in being absorbed by the endocytosis mechanisms or by paracellular diffusion. Although the in-vitro studies suggest that ingestion of the MPs from additively manufactured aligners pose little to no risk in cytotoxicity, the effect of biological environment such as salivary enzymes or the gastrointestinal fluid on the ingested MPs needs to be investigated. The primary finding suggests the safety in using the three aligners for CAT, nevertheless, the reason is open for interpretation through rigorous in-vitro and biocompatibility studies.

## Supporting information

S1 Appendix A (DOCX)

S2 Appendix B (DOCX)
