# Supplementary material for: Experimental assessment of damage and microplastic release during cyclic loading of clear aligners
Source: PLoS One. 2025 Feb 5;20(2):e0318207. doi: 10.1371/journal.pone.0318207 (PMC11798498; doi:10.1371/journal.pone.0318207)
Supplement: S1 Appendix A — (DOCX) [file pone.0318207.s001.docx]

**Appendix B**

Table B1. Dental notations according to ISO 3950 - Dentistry — Designation system for teeth and areas of the oral cavity standard.

| **Upper Left** | | | | | | | | **Upper Right** | | | | | | | |
| --- | --- | --- | --- | --- | --- | --- | --- | --- | --- | --- | --- | --- | --- | --- | --- |
| 18 | 17 | 16 | 15 | 14 | 13 | 12 | 11 | 21 | 22 | 23 | 24 | 25 | 26 | 27 | 28 |
| *Molar* | | | *Premolar* | | *Canine* | *Incisor* | | | | *Canine* | *Premolar* | | *Molar* | | |
| 48 | 47 | 46 | 45 | 44 | 43 | 42 | 41 | 31 | 32 | 33 | 34 | 35 | 36 | 37 | 38 |
| **Lower Left** | | | | | | | | **Lower Right** | | | | | | | |

All the data required to replicate the experiments can be found in the following link.

<https://doi.org/10.6084/m9.figshare.27275268>
